# Analysis of machine perfusion benefits in kidney grafts: a preclinical study

**DOI:** 10.1186/1479-5876-9-15

**Published:** 2011-01-25

**Authors:** Nader Vaziri, Raphaël Thuillier, Frederic D Favreau, Michel Eugene, Serge Milin, Nicolas P Chatauret, Thierry Hauet, Benoit Barrou

**Affiliations:** 1 Inserm U927, Poitiers, Poitiers F-86021, France; Univ Poitiers; Faculté de Médecine et de Pharmacie, Poitiers, F-86034, France; 2 Service d'Urologie et chirurgie de la transplantation, Pavillon V - Hôpital Edouard Herriot - 5, place d'Arsonval, 69437 Lyon, France; 3 CHU Poitiers, Pole UBM, Service de Biochimie, Poitiers, F-86021, France; 4 IBISA, Domaine expérimental du Magneraud, Surgères, F17700, France; 5 Service d'Urologie et Transplantation, Hôpital Pitié Salpétrière, Groupe Hospitalier Universitaire Est, 75651 Paris cedex 13, Paris, France; 6 Université Pierre et Marie Curie, 75005 Paris cedex, Paris France; 7 FLIRT : Fédération pour L'étude de l'Ischémie Reperfusion en Transplantation, Poitiers, F-86034, France

## Abstract

**Background:**

Machine perfusion (MP) has potential benefits for marginal organs such as from deceased from cardiac death donors (DCD). However, there is still no consensus on MP benefits. We aimed to determine machine perfusion benefits on kidney grafts.

**Methods:**

We evaluated kidney grafts preserved in ViaspanUW or KPS solutions either by CS or MP, in a DCD pig model (60 min warm ischemia + 24 h hypothermic preservation). Endpoints were: function recovery, quality of function during follow up (3 month), inflammation, fibrosis, animal survival.

**Results:**

ViaspanUW-CS animals did not recover function, while in other groups early follow up showed similar values for kidney function. Alanine peptidase and β-NAG activities in the urine were higher in CS than in MP groups. Oxydative stress was lower in KPS-MP animals. Histology was improved by MP over CS. Survival was 0% in ViaspanUW-CS and 60% in other groups. Chronic inflammation, epithelial-to-mesenchymal transition and fibrosis were lowest in KPS-MP, followed by KPS-CS and ViaspanUW-MP.

**Conclusions:**

With ViaspanUW, effects of MP are obvious as only MP kidney recovered function and allowed survival. With KPS, the benefits of MP over CS are not directly obvious in the early follow up period and only histological analysis, urinary tubular enzymes and red/ox status was discriminating. Chronic follow-up was more conclusive, with a clear superiority of MP over CS, independently of the solution used. KPS was proven superior to ViaspanUW in each preservation method in terms of function and outcome. In our pre-clinical animal model of DCD transplantation, MP offers critical benefits.

## Introduction

Static cold storage (CS) using the University of Wisconsin solution (Viaspan^®^) (UW) is the gold standard of preservation of kidneys obtained from deceased donors [1]. Its introduction in the late nineteen eighties has reduced the incidence of delayed graft function (DGF) and improved graft survival of kidneys obtained from donations after brain death [2]. Nevertheless, the growing use of expanded criteria donors (ECD), donors with acute renal failure [3,4] and deceased after cardiac death donors (DCD) has increased the DGF incidence of graft preserved by UW [5] or by CS in general [6,7].

Use of DCD grafts in the clinic is limited by a high rate of primary non function and DGF [7-9], in correlation with the length of the warm ischemia period [6]. However, as they represent a significant increase in the pool of donors (30%), which is of particular importance in the current shortage (only one out of three patients on the waiting list receives a kidney), finding the optimal way to preserve these organs and improve their quality as become a first order issue.

Hypothermic machine perfusion (MP) preservation is increasingly being used as an alternative preservation method to CS. Studies have reported a reduction of DGF after MP compared to CS [10-18], however the solutions used were different, and some studies lacked proper randomization. These early clinical data were supported by experimental studies, conducted in large animal models of DCD using different preservation solutions, reporting improvements of kidney function after MP [19-22]. Nevertheless, not all animal studies support the superiority of MP over CS in DCD models. Indeed, MP of pig DCD kidneys using a combination of Belzer machine perfusion solution (MPS) and Viaspan^® ^did not reveal any superior effect to ViaspanUW-CS [20] and when the same preservation solution was used in both the CS- and MP- groups, no significant difference between MP and CS preservation could be observed in dogs [21] or pigs [23] for WI times of up to 60 min. A better performance of ViaspanUW-MP was, however, reported for longer WI times in dogs [21]. These experimental data question the necessity of MP for DCD kidneys. Clinical evidence on the use of MP and its benefits can be conflicting [24-26], however recent clinical trials show small but significant benefits of MP over CS [27] in terms of DGF rate and one year survival of grafts from all categories of donors and further studies demonstrated some benefits from MP in terms of DGF and function in a DCD subset [28].

Hence, clinical evidence for the superiority of MP over CS in DCD kidney transplantation is accumulating and interest in MP is still growing [29-32] as new machines [33,34] and preservation concepts [35] are being developed. Nevertheless there is also a need for preclinical studies in a standardized transplantation model to investigate the benefits of MP on both acute and chronic kidney injury.

The present study uses a recently developed porcine model mimicking conditions of DCD class I and II [36,37], by 60 minutes of WI before organ collection and storage. We propose a four-way comparison using preservation with Viaspan^® ^(ViaspanUW), the gold standard in CS, either by CS or MP, and preservation with Kidney preservation solution-1^® ^(KPS), recommended for MP, either by CS or MP.

We will measure function recovery, quality of function, chronic immune response development, chronic fibrosis development and animal survival. This will allow us to determine a 'machine effect' independently of the solution used, as well as measure benefits of clinical MP (KPS-MP) versus clinical CS (ViaspanUW-CS).

## Methods

### Surgical procedures and Experimental groups

The DCD model was performed in *large white *male pigs (INRA, GEPA, Surgères, France) (30-35 kg) according to the guidelines of the French Ministry of Agriculture for the use and care of laboratories animals as previously described [37]. Briefly, WI was induced by right renal pedicle clamping for 60 min, conditions inducing consistent damages [37]. The right kidney was removed, cold flushed with the same solution used for either MP or CS, and preserved for 24 hours at 4°C either by static storage (CS), or by MP using the Lifeport^® ^machine (Organ Recovery System, USA) with either ViaspanUW (Viaspan^®^, Bristol-Myers Squibb, France) or KPS (KPS-1^®^, Organ Recovery Systems, Brussels). Solution composition is detailed in Table 1. At the end of the preservation period, the kidney is transplanted in the same animal, and the left kidney is removed to reproduce the nephron mass in transplanted patients. Average anastomosis time was 30 ± 5 min and no complications were observed between the 2 surgical procedures.

**Table 1:** Solutions Composition

Componants	Blood	ViaspanUW	KPS
***Ions (mM)***			

Na^+^	140	30	80

K^+^	5	125	25

Mg^2+^	0.8	5	5

Ca^2+^	2.5		0.5

Cl^-^	104		0.5

SO4^2-^	1.4	5	

H_2_PO4^2-^	3.2	25	25

HCO3-	25		

HEPES			10

***Additives***			

Glucose	7		10

Raffinose		30	

Ribose			5

lactobionate		100	

adenosine		5	5

glutathion		4	4

allopurinol		1	

Mannitol			30

***Colloids (g/L)***			

HES		50	50

***Physico-chimie***			

pH	7.4	7.3	7.4

Viscosité (cSt)	1.6	2.4	3.15

Osmolarité (mOsm)	308	320	320

4 groups were studied: **1)ViaspanUW-CS**: kidneys preserved in Viaspan^® ^solution by CS (n = 6); **2)ViaspanUW-MP**: kidney preserved in Viaspan^® ^by MP (n = 8); **3)KPS-CS**: kidneys preserved in CS (n = 7); **4)KPS-MP**: Kidneys preserved in KPS-1^® ^solution by MP (n = 7). Results between experimental groups were compared to a group of normal animals (**Control**; Sham Operated sex-, age- and weight-matched, n = 7).

Primary non-function (PNF) of the graft was defined as a total absence of urine output for 7 consecutive days after transplantation and since dialysis is not available in our animal facility, animals with PNF were sacrificed.

### Organ perfusion parameters

The Lifeport^® ^kidney transporter operated in pulsatile mode, with a maximum systolic pressure set at 40 mmHg and frequency at 60 min^-1^. The initial perfusion pressure was set at 35 mmHg. This setting was corrected hourly, according to the clinical protocol recommended by the "Agence de Biomédecine" (France), based on the organ's value of perfusion resistance (mm Hg/(mL/min) displayed in real time on the machine screen, representing the quotient of pressure divided by flow. The perfusion pressure was corrected according to the 3 following criteria of resistance value: 1) inferior or equal to 0.3 mm Hg/(mL/min), the perfusion pressure setting was decreased at a rate of 5 mmHg/h with a minimal perfusion pressure of 20 mmHg; 2) ranging from 0.3 to 0.6 mm Hg/(mL/min), the perfusion pressure setting was maintained at 35 mmHg; 3) equal or over 0.6 mm Hg/(mL/min), the perfusion pressure setting was increased at a rate of 5 mmHg/h with a maximal perfusion pressure of 45 mmHg. Overall mean pressure was 31.5 ± 2.5 mmHg in the KPS-MP group and 33.4 ± 1.5 mmHg in the ViaspanUW-MP group.

### Functional parameters

Animals were placed in individual metabolic cages for blood and urine collection. Functional parameters were measured using an automatic analyzer (Modular automatic analyzer, Roche Diagnostic, Meylan, France). Activities of brush border enzyme alanine aminopeptidase and lysosomal enzyme *N*-acetyl-β-D-glucosaminidase (NAG) were determined in urine as previously described [38], briefly, NAG activity was determined on a Roche Modular P system (Roche Diagnostics, Meylan, France) and AAP determination was measured using storage method and colorimetric assay. NAG and AAP activity (U/L) was expressed as a ratio with urinary creatinine (mmol/L) so as to adjust for differences in urinary flow of the sample.

### Histopathological studies

Serial ultrason-guided percutaneous biopsies were performed at day 7 and M1 and larger tissue samples were collected at 3 month after sacrifice. Samples were either frozen at -80°C or fixed in formalin then embedded in paraffin. All sections were examined and photographed under blind conditions by a pathologist and a nephrologist. A standard procedure was used to estimate the level of tubulointerstitial fibrosis using the Picro Sirius red staining, as described previously [39]. ED1+ and CD3+ cell invasion was measured on frozen sections from the graft at 3 months, stained with specific antibodies (SouthernBiotech, USA). 10 high powered fields (400X) were randomly selected and the number of positive cells determined in a blinded fashion. Immunostaining was performed for Vimentin (Dako, Sweden). The percentage of staining was determined by computerized image analysis in 10 randomly selected fields (×200) of each slide.

### Statistical methods

Results are shown as mean ± SEM. For the statistical analysis among groups, we used NCSS software (NCSS LLC, USA) an one-way ANOVA analysis with Tukey-Kramer test for multiple comparisons in case of normality (Skewness, Kurtosis and Omnibus tests) and equality of variance (Modified-Levene Equal-Variance Test) and Kruskal-Wallis Multiple-Comparison Z-Value Test (Dunn's Test) in case these parameters were not met. Correlation were evaluated with Pearson and Spearman tests and a 2 way ANOVA test was performed to check influence of preservation techniques and solutions. Statistical significance was accepted for P < 0.05.

## Results

### Organ characteristics

**Figure 1:**
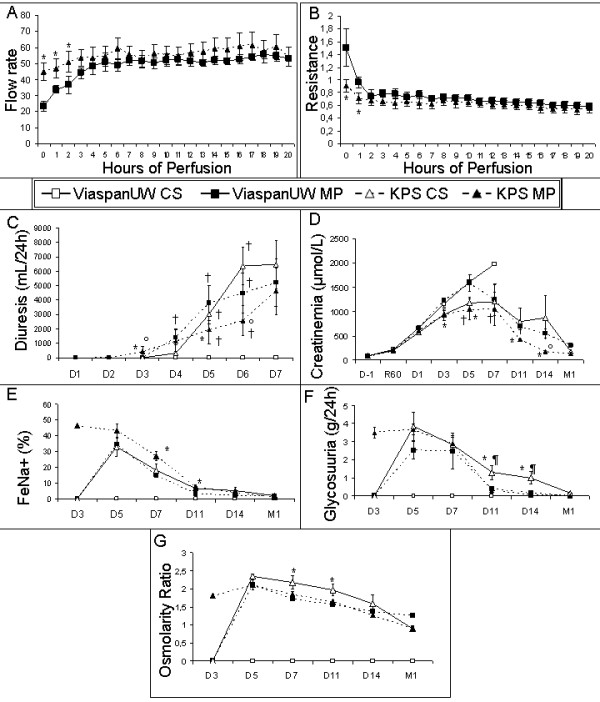
**Perfusion Parameters and Kidney function following reperfusion**. **A**: Flow rate and **B**: Resistance of machine perfused kidneys. **C**: Diuresis before and after transplantation. **D**: Serum creatinine before and after transplantation. **E**: Sodium excretion fraction. **F**: Glycosuria. **G**: Osmolarity ratio between blood and urine. Shown are mean ± SEM, statistics: † : p < 0.05 to ViaspanUW CS; * : p < 0.05 to ViaspanUW MP; ° : p < 0.05 to KPS CS; ¶ : p < 0.05 to KPS-MP.

Kidney's weights before preservation did not differ between the experimental groups (166.9 ± 7.4 g). After preservation, kidneys from ViaspanUW-CS group had lost the most weight (115.0 ± 7.7 g) while KPS-CS kidneys did not change significantly (155.3 ± 13.8 g, p < 0.05 to UW-CS). ViaspanUW-MP organs seemed to gain weight (191.8 ± 16.3 g) while KPS-MP had significantly gained weight (208.6 ± 13.2 g, p < 0.05 to all). Organ resistance was significantly higher and flow rate significantly lower at the start of perfusion for ViaspanUW-MP grafts compared to KPS-MP organs (p < 0.05, Figure 1A and 1B).

### Function recovery (Figure 1C to 1G)

Animals from the ViaspanUW-CS group never recovered diuresis, their serum creatinine increased steadily until day 7 when the obvious lack of function recovery and generally poor state of the animal lead us to euthanize them. ViaspanUW-MP and KPS-CS groups recovered diuresis by day 4 post reperfusion, functional recovery was similar except for a lower creatinine peak at day 5 (p < 0.05) and a higher osmolarity ratio from D5 to D11 for KPS-CS (p < 0.05). KPS-MP demonstrated better function recovery with diuresis resuming at D3, lower serum creatinine levels and a similar osmolarity ratio to ViaspanUW-MP. MP groups also demonstrated controlled glycosuria by D11 (p < 0.05 versus KPS-CS), while glycemia was normal in all groups (data not shown).

### Urinary enzymes (Figure 2 A and 2B)

**Figure 2:**
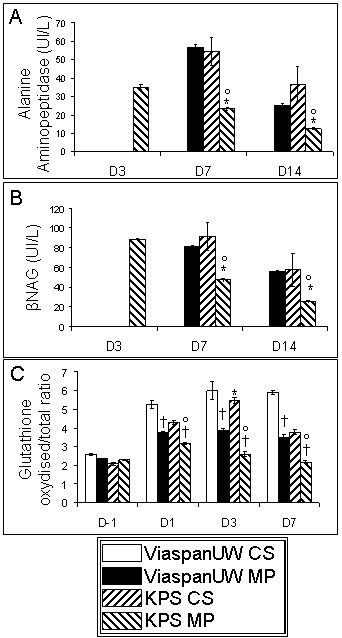
**Tubular integrity and Red/Ox Status following reperfusion**. **A**: Alanine aminopeptidase activity in urine. **B**: β-N-acetylglucosaminidase activity in urine. **C**: Blood reduced gutathion over total glutathion ratio. Shown are mean ± SEM, statistics: † : p < 0.05 to ViaspanUW CS; * : p < 0.05 to ViaspanUW MP; ° : p < 0.05 to KPS CS; ¶ : p < 0.05 to KPS-MP.

Measurement of urinary levels of proximal tubule enzymes alanine peroxydase and *N*-acetyl-β-D-glucosaminidase (β-NAG) showed early high levels followed by a progressive reduction with time, sign of tubular damage and slow recovery of structure. KPS-MP grafts showed fastest and most effective recovery, with ViaspanUW-MP and KPS-CS showing consistently higher levels (p < 0.05).

### Oxydative Stress (Figure 2, C)

Measure in peripheral blood of the ratio of oxidized glutathione over total glutathione, reflecting the oxidative stress state of the animal, showed lowest levels at all time points for KPS-MP group (p < 0.05). ViaspanUW-MP group showed equal or lower levels than KPS-CS. ViaspanUW-CS showed the highest levels for the duration of the follow up. Statistical analysis showed that use of MP was correlated with lower oxidized glutathione levels at Day3 (R^2 ^= 0.76, p < 0.0001) and 2 way ANOVA showed an influence of solution (p < 0.05) and perfusion technique (p < 0.001) while no additive influence was determined. At day 7, MP was also correlated with lower levels (R^2 ^= 0.54, p < 0.01) and 2 way ANOVA showed additive effect of solution and perfusion technique (p < 0.01). Use of KPS was not correlated with lower levels at day 3 while it was slightly correlated with levels at day 7 (R^2 ^= 0.41, p < 0.01)

### Tissue histology (Figure 3, Table 2)

**Table 2:** Histological Evaluation

	ViaspanUW-CS	ViaspanUW-MP	KPS-CS	KPS-MP
***Brush Border loss***				

D7	5.0 ± 0.0	4.1 ± 0.3 †	3.6 ± 0.4 †	3.6 ± 0.5 †

D14	n/a	3.2 ± 0.7	3.0 ± 0.6	2.0 ± 0.4 *

M1	n/a	3.6 ± 0.8	2.0 ± 0.5 *	1.2 ± 0.3 * °

***Endoluminal Detachment***				

D7	5.0 ± 0.0	4.3 ± 0.2 †	3.3 ± 0.3 †	3.0 ± 0.6 †

D14	n/a	3.6 ± 0.8	2.8 ± 0.6	2.0 ± 0.4 *

M1	n/a	2.8 ± 0.6	2.0 ± 0.5 *	1.0 ± 0.2 * °

***Tubulo-interstitial Inflammation***				

D7	necrosis	3.0 ± 0.1	3.0 ± 0.1	2.0 ± 0.1

D14	n/a	3.2 ± 0.2	3.0 ± 0.1	2.0 ± 0.1 *

M1	n/a	2.6 ± 0.3	2.0 ± 0.1	1.0 ± 0.1 * °

Statistics: †:p < 0.05 to UW-CS, *:p < 0.05 to UW-MP, °: p < 0.05 to KPS-CS

**Figure 3:**
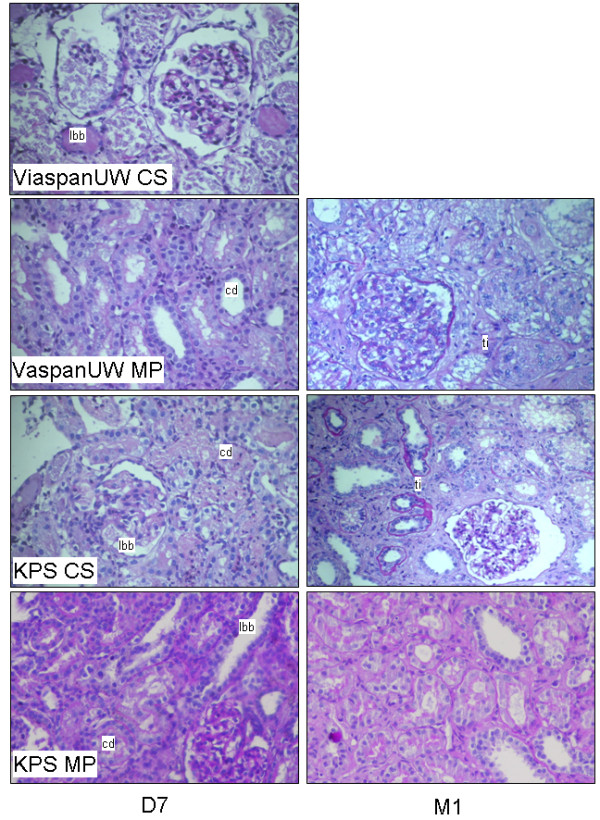
**Graft Histology**. Representative PAS staining of kidney biopsies at day 7 and Month 1 post transplantation. LBB: loss of brush border; CD: Endoluminal cell detachment; Ti: tubulo-interstitial inflammation.

Evaluation of tissue histology at D7 showed intense tissue damage and necrosis for ViaspanUW-CS grafts. There was significantly reduced damage in the ViaspanUW-MP group (p < 0.05) compared to ViaspanUW-CS. KPS grafts tended to show lower amount of damage compared to ViaspanUW kidneys. At D14 and M1, ViaspanUW-MP consistently showed more tissue damage (p < 0.05 at M1) and tubulo-interstitial invasion compared to KPS-CS, and further reduction was observed in KPS-MP kidneys (p < 0.05 to both at M1).

### Immune response development (Figure 4)

**Figure 4:**
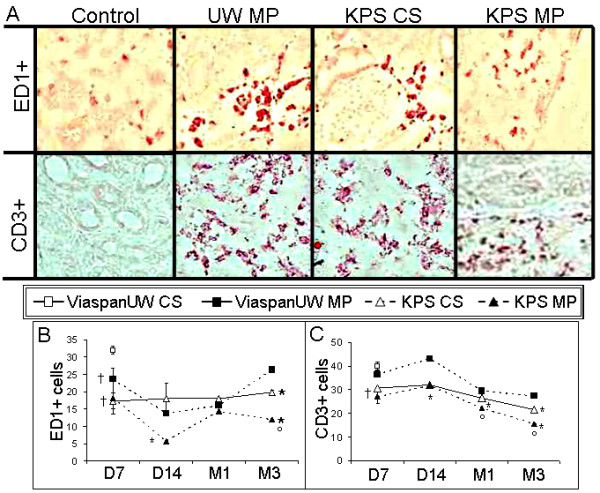
**Inflammation**. **A**: Representative images of typical ED1+ (top) and CD3+ (bottom) staining of kidneys from each group. **B**: graphical representation of the number of ED1 positive cells at each time point for each group. **C**: graphical representation of the number of CD3 positive cells at each time. Shown are mean ± SEM, statistics: † : p < 0.05 to ViaspanUW CS; * : p < 0.05 to ViaspanUW MP; ° : p < 0.05 to KPS CS; ¶ : p < 0.05 to KPS-MP.

Immunostaining for monocyte/macrophages (ED1+) showed consistently lower invasion level in KPS-MP group (p < 0.05), while KPS-CS and ViaspanUW-MP demonstrated similar cell number until M1. After 3 month, invasion in KPS-CS was lower than in ViaspanUW-MP (p < 0.05). Staining for CD3+ showed lower levels in KPS groups compared to ViaspanUW groups throughout the duration of the follow up (p < 0.05). KPS-MP grafts had lower invasion levels compared to KPS-CS starting from M1 until M3 (p < 0.05). Use of KPS was correlated with lower invasion lovels for both ED1+ (R^2 ^= 0.75, p < 0.0001) and CD3+ (R^2 ^= 0.78, p < 0.0001). Within the KPS groups, MP was correlated with lower invasion (ED1+: R^2 ^= 0.96, p < 0.0001; CD3+: R^2 ^= 0.98, p < 0.0001)

### Epithelial to Mesenchymal Transition (Figure 5)

**Figure 5:**
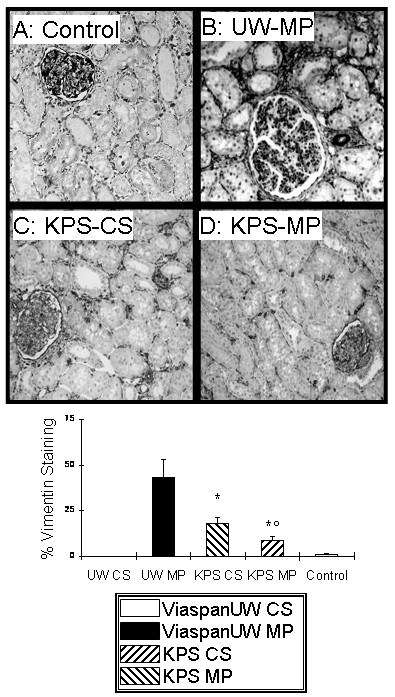
**EMT development**. **A, B, C and D**: Representative staining for Vimentin at 3 months. E: quantification of staining in each group. Shown are mean ± SEM, statistics: † : p < 0.05 to ViaspanUW CS; * : p < 0.05 to ViaspanUW MP; ° : p < 0.05 to KPS CS; ¶ : p < 0.05 to KPS-MP.

Evaluation of Vimentin staining at 3 month revealed high levels of Vimentin expression in ViaspanUW-MP kidneys. Expression was halved in KPS-CS kidney (p < 0.05) and further diminished in KPS-MP grafts (p < 0.05 to both KPS-CS and ViaspanUW-MP).

### Renal survival, Function and Interstitial Fibrosis/Tubular Atrophy (Figure 6)

**Figure 6:**
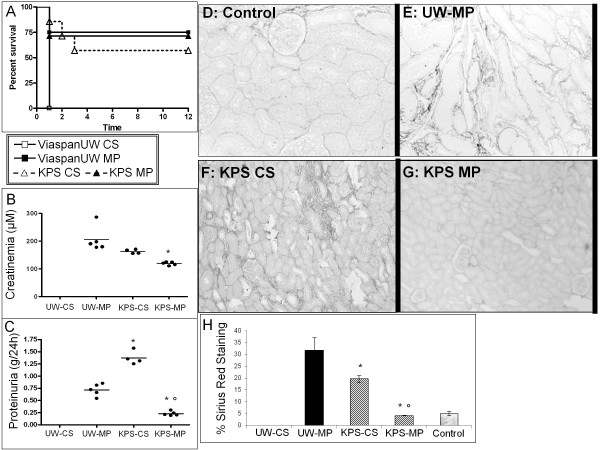
**3 month Outcome**. Survival was measured and represented by a Kaplan-Meier plot (**A**). Function was determined: Creatinemia (**B**) and proteinuria (**C**) Representative images of Sirius Red staining of sections obtained from Control (**D**), ViaspanUW-MP (**E**), KPS-CS (**F**) KPS-MP (**G**) kidneys. Original magnification x100. **H**: Quantification of fibrosis in kidneys from each group studied. Shown are mean ± SEM, statistics: † : p < 0.05 to ViaspanUW CS; * : p < 0.05 to ViaspanUW MP; ° : p < 0.05 to KPS CS; ¶ : p < 0.05 to KPS-MP.

No animal of the ViaspanUW-CS group survived beyond D7. Three months after transplantation, survival was lowest in KPS-CS group, followed by KPS-MP with ViaspanUW-MP showing the highest survival rate, although the differences were not significant. Morphological analysis (Additional file 1) revealed extensive necrosis and tubule loss at week 1 for cases of primary non function (PNF), graft loss at weeks 2 and 4 was due to high rate of inflammation and tubulitis. Serum creatinine was highest in ViaspanUW-MP group, followed by KPS-CS (p < 0.05) and KPS-MP (p < 0.05 to both).

This order was also found when evaluating fibrosis development: ViaspanUW-MP kidneys showed more than 30% fibrosis, while KPS-CS neared 20% (p < 0.05 to ViaspanUW-MP). Fibrosis development in KPS-MP was negligible and did not differ from control. Here also, use of KPS correlated with lower fibrosis (R^2 ^= 0.65, p < 0.01). Within the KPS groups, MP was correlated with lower fibrosis (R^2 ^= 0.87, p < 0.01).

## Discussion

Herein, we demonstrate in a preclinical study using a highly reproducible swine model of transplantation the benefits of MP over CS, particularly in regards to chronic outcome.

We performed static preservation with both ViaspanUW and KPS, demonstrating the superiority of KPS in terms of function recovery, histology at D7 and survival. Comparisons of these two groups offers a perspective on studies generally performed on machine perfusion: when two different solutions are used for static and machine preservation, the observed effect is not solely due on perfusion but also depends significantly on the solution used. Our 4 groups/2 variables approach circumvents this bias, highlighting the importance of animal studies in large animals to assess the benefits of novel therapies, as indeed such setting is impossible in the clinic.

Weight variations of kidney grafts are classically observed during preservation. Our observation of weight loss for CS and weight gain for MP are consistent with a similar experimental design in pigs [23]. In addition, increases in kidney weight after MP have been previously reported to have no significant impact on the graft outcome [40].

Comparing ViaspanUW-CS to ViaspanUW-MP allows us to determine the benefits of machine perfusion with the current high-K^+ ^gold standard in static preservation. Although ViaspanUW is not used for MP in clinical settings, using identical preservation solution focuses the analysis solely on the effect of perfusion. Early follow up with classical tools such as serum creatinine do not allow to determine differences between the two methods. In our setting, pigs were not dialyzed thus analysis of diuresis was pertinent, but this would not be the case in the clinic. Interestingly, measure of peripheral blood gluthathion red/ox status provided discriminating information between the groups, which was enhanced by analysis of histology at day 7. Use of UW demonstrates in the clearest fashion the benefits of MP: while high concentration of potassium induce vasoconstriction, as seen in the resistance index at beginning of perfusion, the machine is able to rescue this negative effect and regulate flow, allowing the organ to better face the stress of reperfusion, with dramatic benefits on outcome particularly survival, such as found in the clinic [27,28]. This model thus offers a unique opportunity for further clarification of the exact mechanisms through which MP provides this protection.

Benefits of machine perfusion were also not immediately obvious between KPS-CS and KPS-MP groups: diuresis and creatinine levels were close, as were other functional parameters usually available in the clinic. Here also, discrimination was possible with measure of Glutathion red/ox. Moreover, since both groups produced urine, proximal tubule enzymes activity assay in the urine was invaluable. Alanine aminopeptidase and β-N-acetylglucosaminidase are found in kidney tubular cells brush border and their presence in urine is a commonly accepted sign of tubular damage [41], their activity level in the urine revealed a superiority of MP in maintaining tissue integrity at all time point, which was confirmed by histological analysis of the grafts parenchyma.

Early follow up of ViaspanUW-MP and KPS-CS showed similar values on the tests we performed, highlighting the existence of a solution bias when comparing preservation strategies. Altogether, results from the early follow up do not permit a clear discrimination between CS and MP, unless we consider less orthodox tests such as glutathione red/ox or urinary tubular enzyme activity assays. Excretion of Na^+ ^and glycosuria, in a context of normoglycemia, also offered a degree of discrimination between experimental groups for tubular necrosis and tubular dysfunction.

In the case of glutathione red/ox, a clear correlation was drawn between the use of MP and lower oxidative stress, and both solution and perfusion technique demonstrated an effect on this parameter. However, addition of effects was not found until day 7. We thus identify an independent machine effect, however the relatively small differences observed herein would likely not be present in the clinic due to disparities in patients population, while in identical pigs statistical significance is obtainable. Grafts histology analysis confirmed the superiority of MP over CS, however these tests may not be standard in clinical practice. Thus, measurement of the benefits of MP is difficult in short follow up studies, particularly if the preservation solution bias is not circumvented.

We followed animals for 3 month post reperfusion. In this large animal model, this length permits us to follow the development of chronic lesions such as immune response and interstitial fibrosis and tubular atrophy (IFTA). The summated effects of damage sustained by organ preservation and reperfusion [42] lead to loss of graft function, and ultimately loss of the grafts itself, often due to the development of IFTA [43]. This pathology is also strongly correlated with immune response [42,44-46]. Herein, KPS-MP showed less innate and adaptative invasion compared to KPS-CS, which showed lower levels that ViaspanUW-MP. Use of KPS correlated with lower invasion, and within the KPS groups we showed that the use of MP correlated with better outcome. Unfortunately, absence of data from the UW-CS group did not allow us to perform further statistical analysis. This confirms the benefits of the machine on chronic immune response development. The ViaspanUW-MP fared poorly compared to KPS groups, however its superiority to ViaspanUW-CS is demonstrated in terms of animal survival. These results are in contradiction to a study conducted on dogs [21], however the setting of the study and the anatomy of the dog kidney render the comparison of data difficult.

Epithelial to mesenchymal transition (EMT), a process through which polarized tubular cells are driven to de-differentiate and alter their phenotype towards that of a mobile and fast proliferating mesenchymal cell [47], is shown to be a repair mechanism that can be deregulated during injury and promote interstitial fibrosis [48-50]. Our results show that Vimentin staining, a marker of EMT, is high in ViaspanUW-MP, lower in KPS-CS and close to control levels in KPS-MP. Thus, the machine effect is also found in a major pathway leading to fibrosis and graft loss. We measured the extend of fibrosis using Sirius red and showed a similar order in the grade of lesion: ViaspanUW-MP was highest and KPS-CS showed half the degree of fibrosis of ViaspanUW-MP. KPS-MP group did not show a degree of fibrosis higher than control.

Considering no ViaspanUW-CS animal survived to the end of the follow up, no comparison is possible in regards to chronic lesions such as immune response or fibrosis, however previous studies using the same protocol as ViaspanUW-CS showed a 27% survival rate with important immune response and IFTA (47%) [51,52]. ViaspanUW-MP showed better survival, strengthening the results of a similar study investigating the short-term effects (7 days) of ViaspanUW-MP in a pig model [19], also reporting trends towards a better early kidney function after MP [19,23]. Our results demonstrate superiority of KPS over ViaspanUW solution in our animal model, independently of the preservation strategy. UW is a high K^+ ^and low Na^+ ^solution [53], proposed to maintain intracellular ionic balance. However high potassium has been shown to induce cellular depolarization, decrease cellular ATP content and activates voltage-dependent channels, such as calcium channels [54,55]. Influx of calcium can result in vasoconstriction impairing organ perfusion during washout and reperfusion, participating in the 'no reflow' phenomenon [56-58]. Recently, studies have shown equal or improved results of low potassium/high sodium ratio such as KPS [1], consistent with our findings. Use of Mannitol instead of lactobionate in KPS may also account for the better performance, as this compound has reactive oxygen species scavenging properties [1].

The present study uses *large white *pigs, an animal well suited for preclinical studies as it is close to humans, particularly in regards to the multipapillar multilobular organization of its kidney, only found in higher mammals, implying a complex vascular bed making these organs particularly sensitive to IRI [59]. In this setting, we determined that the benefits of machine perfusion, with a machine currently used in the clinic, are most evident on chronic graft outcome. Indeed, discrimination between the groups in the early time points was only possible through assays rarely performed in transplant centers and thus could explain the relatively small benefits found in clinical studies investigating the machine effect [27]. However, our results suggest that chronic follow up of these patients will uncover a wider rift between MP and CS, as chronic lesions start to develop.

The exact mechanisms by which MP minimizes the activation of lesional pathways in our study remain to be elucidated. MP actions may include a complete perfusion of the organ promoting a thorough washout of blood and subsequent tissue equilibration with the preservation solution. This more efficient washout has been previously reported to limit the aggregation of erythrocytes [60]. Finally, the maintenance of a flow may protect against depolarization of the endothelial cell membrane which is linked to generation of ROS, increased intracellular Ca^2+ ^concentration, and activation of NO synthases [61]. Hence, more mechanistic studies are necessary to unravel the exact mechanism of action in MP, in order to focus on improvement and optimal application of this technique.

The present study appears limited by the use of an isograft model, devoid of the influence of immunosuppressants. However, machine perfusion has been developed to optimize graft preservation, hence address ischemia reperfusion injury. Thus, we felt that an allograft model, with the addition of immunosuppressors and their own set of deleterious side effects, would dilute the impact of our results. We thus sacrificed relevance to the clinic by the use of isograft in order to obtain clarity of our results in regards to the benefits of machine perfusion. Another limitation is the fact that our model does not follow exactly the setting of classes I and II of the Maastricht criteria. Indeed, it normally includes no more than 30 min arrest before starting the CPR procedure, which is then continued during the transport to the hospital (generally with a machine); then as failure to resuscitate is pronounced there is a 5 min no touch period. All these steps should not exceed 150 min. The patient is then either cold perfused or a extracorporeal circuit is put in place, giving enough time to secure consent from the family and collect the organs, which are then machine perfused. It is obvious that a correct modelling of this situation should include all these steps, and we are actually in the process of adapting such procedures on the pig. However in the meantime we are using 60 min WI as it reproduces as closely as possible the conditions of DCD.

## Conclusion

In a study using a preclinical model of DCD kidney transplantation, we demonstrate the superiority of MP over CS independently of the solution used for perfusion. Our results suggest significant benefits on graft outcome, particularly evident on the chronic effects of IRI with a protection against chronic immune response, EMT and IFTA.

## Competing interests

The authors declare that they have no competing interests.

## Authors' contributions

NV carried out the animal experiment design, surgery, data gathering. RT carried out the data management and processing, writing the paper. FF carried out experimental design, running experiments, data gathering. NC carried out the experiments, data gathering. SM carried out the experimental design, running experiments, data gathering. ME carried out the study design. TH carried out the study design, writing the paper. BB carried out the animal experimental design, study design. All authors read and approved the final manuscript.

## Supplementary Material

Additional file 1 **Representative graft morphology for kidney lost during follow up**. Morphological analysis of grafts lost during follow up revealed extensive necrosis and tubule loss at week 1 for cases of primary non function (PNF). Graft loss at weeks 2 and 4 was due to high rate of inflammation and tubulitis. Click here for file
