# Supplementary material for: Analysis of machine perfusion benefits in kidney grafts: a preclinical study
Source: J Transl Med. 2011 Jan 25;9:15. doi: 10.1186/1479-5876-9-15 (PMC3038164; doi:10.1186/1479-5876-9-15)
Supplement: Additional file 1 — Representative graft morphology for kidney lost during follow up. Morphological analysis of grafts lost during follow up revealed extensive necrosis and tubule loss at week 1 for cases of primary non function (PNF). Graft loss at weeks 2 and 4 was due to high rate of inflammation and tubulitis. [file 1479-5876-9-15-S1.PPT]

## Slide 1
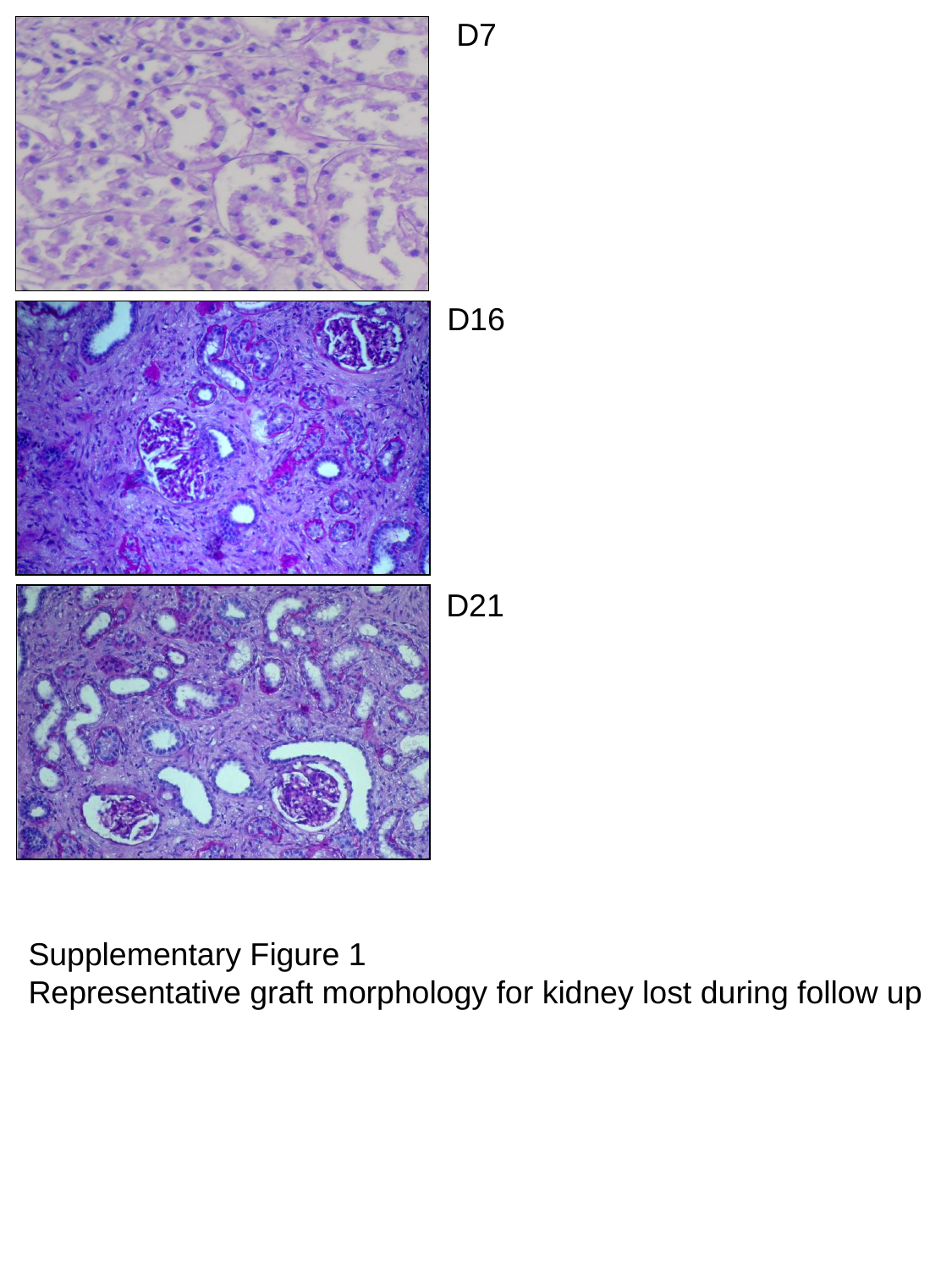

D7
UW CS
D16
D21
Supplementary Figure 1
Representative graft morphology for kidney lost during follow up
